# Medicare Reimbursement Trends for Mandibular Fracture Repair, 2000–2024

**DOI:** 10.1002/lary.70084

**Published:** 2025-08-26

**Authors:** Om B. Tripathi, Aman M. Patel, Christopher C. Vanison

**Affiliations:** ^1^ Department of Otolaryngology Rutgers New Jersey Medical School Newark New Jersey USA; ^2^ Department of Otolaryngology‐Head and Neck Surgery Renaissance School of Medicine at Stony Brook University Stony Brook New York USA

**Keywords:** facial fractures, mandible, Medicare, otolaryngology, reimbursement

## Abstract

**Objective:**

Medicare physician fee schedule (MPFS) reimbursement, historically subjected to complex policy changes, has decreased in numerous surgical specialties after inflation adjustments. Outpatient prospective payment system (OPPS) reimbursement has largely been uninvestigated. This study aims to evaluate monetary trends in Medicare physician and hospital outpatient reimbursement rates for mandibular facial fracture repairs from 2000 to 2024.

**Methods:**

The Physician Fee Schedule Tool and Quarterly Addenda Updates provided by the Center for Medicare and Medicaid Services (CMS) were queried for the six most common open mandibular facial fracture procedures. Physician reimbursement rates for facility and non‐facility clinical settings, hospital outpatient reimbursement rates, work relative value units (RVUs), and total billed services pertaining to these six procedures were collected and analyzed. Monetary data was adjusted for inflation to reflect 2024 dollars based on the consumer price index (CPI) released by the US Department of Labor's Bureau of Labor Statistics.

**Results:**

Average facility physician reimbursement, after adjusting for inflation, of the six mandibular fracture procedures from 2000 to 2024 decreased by 24.1%. All procedures continuously billed in non‐facility settings exhibited physician reimbursement increases, even after post‐inflation adjustments. Hospital outpatient reimbursement, after adjusting for inflation, from 2004 to 2024 increased by 110.6%.

**Conclusion:**

Facility physician reimbursement for mandibular fractures has declined over the past two decades despite increasing hospital compensation and work RVUs. Future reform should incorporate solutions to address declining facility physician compensation in order to mitigate financial strain on providers and barriers to patient access to care.

## Graphical abstract

This study evaluates Medicare reimbursement trends for open mandibular facial fractures procedures from 2000 to 2024. Our results highlight a 24.1% inflation‐adjusted decrease in payments for these procedures, illuminating the need for appropriate funding and resources for physicians. This will help mitigate financial strain for providers and allow for more physician participation in offering care to Medicare patients, especially in light of a growing geriatric population.

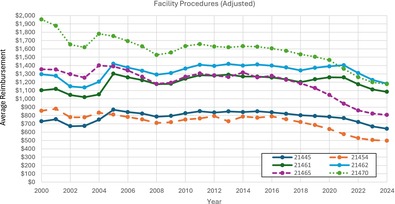

## 1 Introduction

The Centers for Medicare and Medicaid Services (CMS) provides insurance to residents of the United States of America who are 65 and older or have a disability, end‐stage renal disease, or amyotrophic lateral sclerosis. Medicare is one of the largest payors in the United States, insuring over 66 million individuals [1]. CMS provides compensation to physicians using the Medicare Physician Fee Schedule (MPFS), which standardizes payments for any given procedure or service defined by a *Current Procedural Terminology* (CPT) code. MPFS is based on a complex set of factors that CMS annually updates to maintain federal budget neutrality and reflect changes in medical practice costs and efforts. These factors consist of three components: relative value units (RVU), geographic practice cost index (GPCI), and a conversion factor that is updated annually with congressional approval. RVUs are assigned to a CPT code and then adjusted by the GPCI and multiplied by the conversion factor [2]. Beyond physician compensation, CMS also offers reimbursement in outpatient hospital settings through its Outpatient Prospective Payment System (OPPS). OPPS methodology primarily consists of grouping services into Ambulatory Payment Classifications (APCs) based on clinical similarity and resource usage [3].

In an effort to increase transparency, CMS has made reimbursement data publicly available and releases annual updates [4]. As a result, Medicare payments have fallen under greater scrutiny in the past decade, with concerns emerging regarding the inability of facility physician reimbursement to keep up with rising practice costs and inflation. Although the Medicare budget accounts for these factors, increasing spending in one area must be offset by cuts in another. This, in turn, has been shown to negatively impact facility physician reimbursement in surgical specialties such as otolaryngology [5]. Previous studies characterizing Medicare physician reimbursement trends of otolaryngology and its subspecialties have shown declining facility physician reimbursement relative to inflation [6, 7, 8, 9, 10, 11, 12, 13]. However, facility and non‐facility physician reimbursement trends for mandibular fracture procedures remain largely unstudied.

An analysis of reimbursement trends of mandibular fracture procedures holds important significance, as mandibular fractures are common, especially in traumatic settings where patients require emergent, time‐sensitive care. Unlike other types of facial fractures that may not require surgical intervention, mandible fractures often require operative repair because of the complexity of their surrounding anatomy and function [14, 15]. In addition, advanced age has been associated with increased early postoperative complications following open mandibular fracture repair [16]. Inevitably, this places a significant financial burden on hospitals and surgeons and may further disincentivize practitioners to accept Medicare patients if reimbursements are not aligned with the cost and complexity of care. Very little Medicare trend analysis has focused on facial fractures, and none, to our knowledge, on mandibular fractures, particularly for recent years and across different clinical settings (i.e., facility and non‐facility) [17]. Moreover, a lack of significant attention has been paid to reimbursement trends for outpatient hospital department services. This is particularly relevant to mandibular procedures, as recent studies have increasingly supported the safety of orthognathic surgeries in outpatient settings, highlighting the need to understand the evolving financial landscape of OPPS [18, 19].

Our temporal study of Medicare payments aims to inform healthcare policy and provide a comprehensive evaluation of reimbursement patterns for open mandibular fracture repairs. By reporting data on each CPT code related to open mandibular fracture repair, we hope to provide a more granular analysis of reimbursement patterns; additional understanding of economic trends related to these procedures is particularly important because utilization of these codes in recent decades has been impacted by advances in surgical technology and techniques.

## 2 Materials and Methods

Six of the most common open mandibular fracture repair procedures were selected, and their corresponding CPT codes were identified (Table 1). CMS data were accessed online through the publicly available MPFS tool [20]. MPFS consists of reimbursement rates for two types of locations: facility settings, primarily hospitals or ambulatory surgical centers, and non‐facility settings (only if the complete procedure is performed there), primarily physician offices or home services. Both types of physician reimbursement rates were gathered for each procedure from 2000 to 2024. Reimbursement values were averages across all Medicare Administrative Contractor (MAC) localities across the United States. Work RVUs were also collected. Next, a similar analysis focusing on Medicare's direct payment to hospital outpatient facilities was also conducted. Quarterly Addendum B Updates released by CMS from 2004 (the oldest publicly available data) to 2024 were accessed. Annual OPPS payments for the six CPTs were calculated as quarterly averages [21].

**TABLE 1:** Mandibular facial fracture CPT code summaries for open treatments.

CPT Code	Description
21445	Open treatment of mandibular or maxillary alveolar ridge fracture (separate procedure)
21454	Open treatment of mandibular fracture with external fixation
21461	Open treatment of mandibular fracture; without interdental fixation
21462	Open treatment of mandibular fracture; with interdental fixation
21465	Open treatment of mandibular condylar fracture
21470	Open treatment of complicated mandibular fracture by multiple surgical approaches including internal fixation, interdental fixation, and/or wiring of dentures or splints

To standardize comparison over time, physician and hospital reimbursement rates were adjusted for inflation in United States 2024 dollars by querying the US Department of Labor's Bureau of Labor Statistics for the latest available CPI data [22]. Graphical depictions of work RVUs, number of total billed services, unadjusted non‐facility, adjusted non‐facility, unadjusted facility, and adjusted facility physician reimbursement trends from 2000 to 2024, and unadjusted and adjusted outpatient hospital reimbursement from2004 to 2024 were constructed. Total billed services, defined as the number of services performed for a specific Part B procedure minus the denied services, were collected from the CMS Part B National Summary Data Files between 2000 and 2021, the latest available data at time of collection [23]. Adjusted facility reimbursement percentage changes between 2000 and 2024 were stratified by CPT (Table 2). Data was summarized and visualized through Microsoft Excel.

**TABLE 2:** Percent changes in adjusted physician facility reimbursement rates for selected CPT codes between 2000 and 2024.

CPT Code	Adjusted reimbursement rate in 2000 (in 2024 dollars)	Adjusted reimbursement rate in 2024 (in 2024 dollars)	Total adjusted % change
21445	729.50	641.29	−12.09%
21454	856.76	496.66	−42.03%
21461	1102.30	1084.36	−1.63%
21462	1295.38	1181.33	−8.80%
21465	1354.29	806.45	−40.45%
21470	1953.94	1176.88	−39.77%
Average			−24.13%

## 3 Results

**FIGURE 1:**
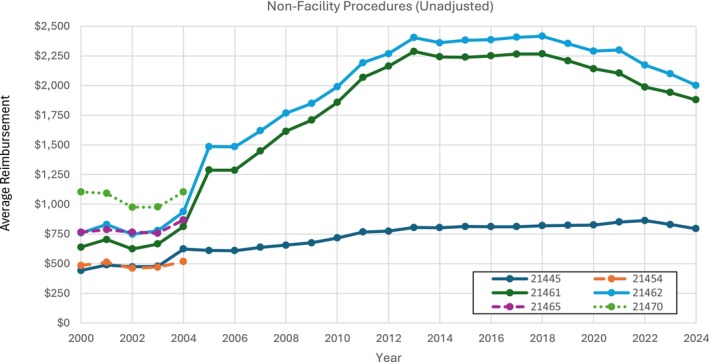
Unadjusted non‐facility physician procedures for open mandibular fracture procedural codes. [Color figure can be viewed in the online issue, which is available at www.laryngoscope.com]

**FIGURE 2:**
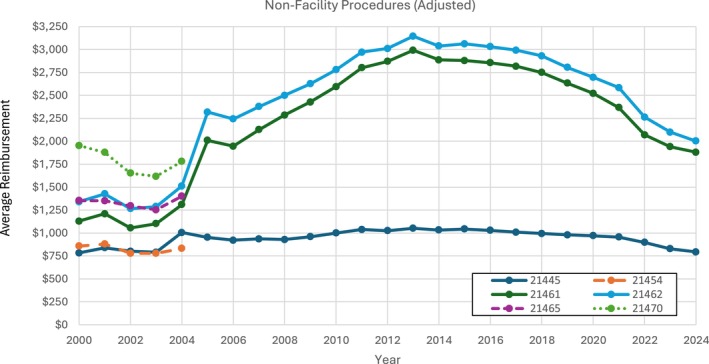
Unadjusted facility physician procedures for open mandibular fracture procedural codes. [Color figure can be viewed in the online issue, which is available at www.laryngoscope.com]

Only CPT codes 21445, 21461, and 21462 were billed continually for non‐facility physician reimbursement between 2000 and 2024 (Figures 1 and 2). Among these three procedures, all rose in reimbursement value, and CPT code 21461 saw the largest unadjusted increase (194.4%). The other three procedures were billed only from 2000 to 2004 and showed stable to small rise in reimbursement value, with CPT code 21465 showing the largest increase (13.4%). When adjusted for inflation, non‐facility physician payments for all three continuously billed procedures rose, but to a much lesser extent (Figure 2). For 2000 to 2004 billed procedures, CPT code 21465 still showed a rise in reimbursement post‐adjustment (3.41%) while the others decreased.

**FIGURE 3:**
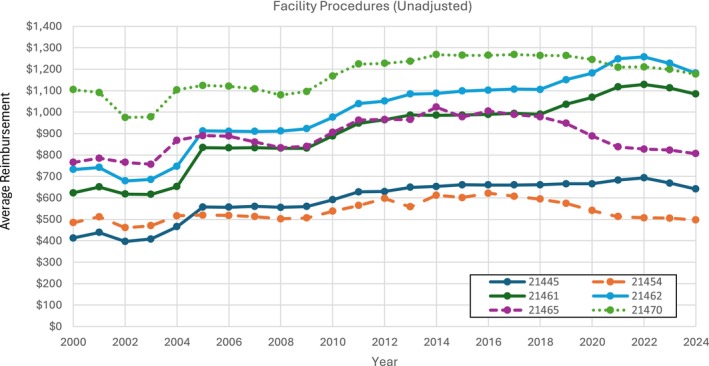
Adjusted non‐facility physician procedures for open mandibular fracture procedural codes. [Color figure can be viewed in the online issue, which is available at www.laryngoscope.com]

**FIGURE 4:**
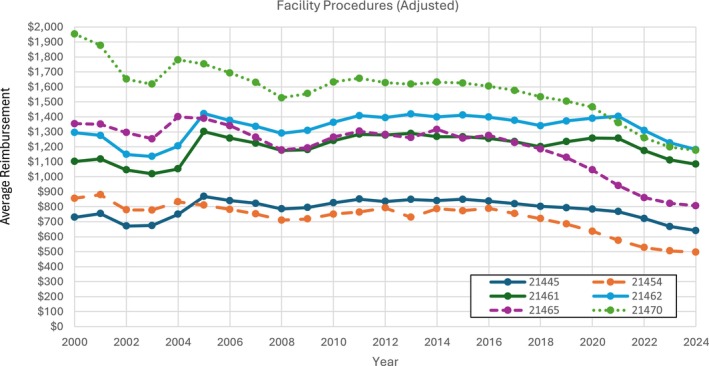
Adjusted facility physician procedures for open mandibular fracture procedural codes. [Color figure can be viewed in the online issue, which is available at www.laryngoscope.com]

For facility physician reimbursement, all procedures evaluated had annual billing throughout the 24‐year period (2000–2024). CPT code 21461 displayed the highest unadjusted rise in reimbursement rate (74.1%) followed by codes 21462 (61.4%) and 21445 (55.6%) (Figure 3). Yet, after inflation adjustments, every procedure's facility physician reimbursement had an overall decrease from 2000 to 2024 (Figure 4). In fact, the six CPT codes showed an average facility physician reimbursement rate decrease of 24.1%, with the largest decrease sourcing from CPT code 21454 (−42.0%) closely followed by CPT codes 21465 (−40.5%) and 21470 (−39.8%) (Table 2). Adjusted yearly percent changes for all procedures showed an average decrease of 1.12% per year (Table S1).

For hospital outpatient reimbursement, CPT codes 21462, 21465, and 21470 were bundled into the same APC by CMS throughout the 20‐year period (2004–2024), and hence saw an identical unadjusted reimbursement rise of 190.9%, translating to an adjusted rise of 75.2% (Figure S1). CPT codes 21445 and 21451 were initially bundled into a different APC from the other four procedures until 2013 and 2014, respectively. They saw an identical unadjusted hospital outpatient reimbursement rise of 367.2%, translating to an adjusted rise of 181.3% (Figure S2). The six CPT codes showed an average hospital outpatient reimbursement rate increase of 110.6% (Table S2). Adjusted yearly percent changes for all procedures showed an average increase of 4.11% per year.

Work RVUs for each procedure increased by over 10%, with CPT code 21445 showing the largest overall rise (16.36%) (Figure S3). All procedures had a notable rise in RVU value in 2007 and then continued to plateau until 2024.

**FIGURE 5:**
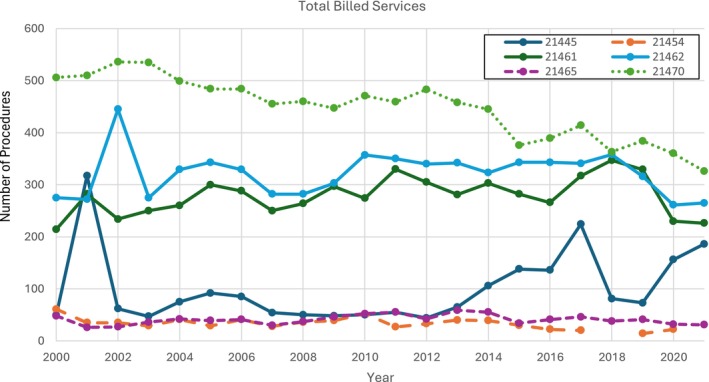
Total billed services for open mandibular fracture procedural codes. Services data for current procedural terminology (CPT) 21454 was missing in 2018 due to a value less than 11 that the Center for Medicare a Medicaid Services (CMS) screened for privacy. [Color figure can be viewed in the online issue, which is available at www.laryngoscope.com]

Lastly, total billed services data from 2000 to 2021 were continuously collected for all CPTs, except 21454, which had “N/A” delineated in the files for 2018 and 2021, indicating a value of less than 11 that had been screened for privacy. The other CPT codes (21445, 21461, 21462, 21465, 21470) changed in number of billed services from 2000 to 2021 by 279.6%, 5.61%, −3.64%, −35.4%, and −36.57%, respectively. Most procedures had periods of volatility throughout the 21‐year period, particularly CPT code 21445 (Figure 5).

## 4 Discussion

Medicare physician reimbursement remains complicated and controversial, even addressed within the 2024 Medicare Trustees Report, stating “certain features of the current law [for reimbursement]” will have longstanding and challenging implications for provider participation and access‐to‐care [24]. As the otolaryngologic geriatric patient population is projected to grow from 17.9% in 2010 to 30% by the year 2030, understanding reimbursement policies is increasingly vital [25]. A 2024 study by Haglin et al. showed correlations between decreased reimbursement and a sicker Medicare population for arthroscopy patients, suggesting the detrimental impact lower reimbursement may have on the quality and access‐to‐care [26, 27]. In fact, the National Bureau of Economic Research conducted a study analyzing the effects of increasing Medicaid reimbursement payments by $10. They found a 25% decrease in parents not finding a doctor for their children covered by Medicaid, along with a 14% decrease in chronic absenteeism due to illness or injury [28]. While directly relevant to publicly funded health insurance, private payors are also influenced by CMS policies [29]. Hence, it is important for otolaryngologists to understand Medicare reimbursement trends within their field to ensure they are well‐equipped to serve their patient population.

Our study investigates Medicare reimbursement trends and utilization for six open mandibular fracture procedures. Average inflation‐adjusted facility physician reimbursement decreased 24.1% in facility physician reimbursement from 2000 to 2024, despite work RVUs increasing for each procedure (Table 2). Moreover, all six procedures had a lower monetary facility physician reimbursement value in 2024 than in 2000 (Table 2). While we only focused on mandibular fractures, these results are consistent with findings of average facility physician reimbursements for all surgical facial fracture repairs, which showed a fall of 44.1% from 2000 to 2019 [17]. This adjusted decline in Medicare facility physician reimbursement has also been witnessed in a wide variety of other otolaryngologic subspecialties: 37.6% decrease from 2000 to 2019 in the 20 most common otolaryngologic procedures [30], 50.0% decrease in low‐wRVU rhinology and 36.1% decrease in high‐wRVU rhinology from 2000 to 2021 [5], 27.5% decrease in pediatric otolaryngology from 2000 to 2020 [31], 20.3% decrease in laryngology from 2000 to 2021 [32], 21.2% decrease in otology from 2000 to 2020 [33], and 19.4% decrease in head and neck surgical oncology from 2000 to 2020 [34].

Notably, our decrease of 24.1% for mandibular facial fractures is relatively lower than average facility physician reimbursement changes in all other otolaryngologic subspecialties from the past decade [35]. This may be due to our exclusion of closed procedures, which have previously been shown to exhibit large Medicare facility physician reimbursement drops over time. For example, Kandi et al. conducted a study for the top 20 surgical procedures in craniofacial trauma and found closed treatment of temporomandibular joint dislocation and closed treatment of nasal bone fractures without manipulation demonstrated 48.7% and 48.3% decreases, respectively. Additionally, despite a similar study for all facial fracture procedures finding a 44.1% fall in average Medicare payments, it was advised to be interpreted with caution since this value disproportionately included lower reimbursements from bone/septum reductions, procedures that are primarily closed approach [17].

Incidence rates of open mandibular procedures between 2000 and 2021 were relatively stable for most CPT codes, aligning with a previous finding of Medicare‐billed mandibular fracture operations only decreasing by 3.2% between 2000 and 2019 [17]. This may be explained by elderly patients undergoing less operative management compared to younger adults to avoid long recovery times and unfavorable outcomes [36, 37]. A shift toward closed reductions in facial fracture repairs, marked primarily by nasal bone/septum operations, explains why facial fracture repairs have increased over time despite open procedure cases displaying little variation. The debate over surgeons utilizing open or closed procedures is still widely a topic of conversation, and an increase in closed repairs may be attributed to the increased comfortability and knowledge of this type of operative procedure, particularly for the nose [17, 38, 39, 40].

Incidence rates for CPT 21470 (*open treatment of complicated mandibular fracture by multiple surgical approaches including internal fixation, interdental fixation, and/or wiring of dentures or splints*) showed a steady decline, possibly explained by evolving surgical techniques that have reduced the need for multiple surgical approaches [41]. In stark contrast, CPT 21445 (*open treatment of mandibular or maxillary alveolar ridge fracture*) showed an overall increase in total billed services by nearly 280%. Computed tomography (CT) imaging is considered the gold standard for assessing alveolar fractures, and improvements in its detection capabilities over the past two decades, bundled with increased fall risk in an aging population, could be aspects for this procedure's increase [42, 43]. Regardless, explanations should be interpreted with caution because this procedure experienced the largest increase in work RVU over time (16.36%); clinical overlap with other procedures in cases of mild mandibular fractures, along with physician billing preferences to optimize reimbursement, may confound a true increase in incidence.

Interestingly, all three continuously billed non‐facility procedures increased in reimbursement, even after inflation adjustments, which is unlike other otolaryngologic subspecialties that witnessed declines in non‐facility reimbursements over time [32, 44]. This may reflect MPFS “global” methodology, which generally reimburses non‐facility procedures at higher rates to cover the physician's labor and all overhead costs [44, 45]. Still, more work is needed in non‐facility physician reimbursement trends, as previous literature has urged investigation to provide a more comprehensive reimbursement trend overview and to offer context to recent site‐neutrality policies and service locality debates [30, 32].

Regardless, our findings for the overall decrease in facility physician reimbursements have significant consequences for physicians and patients alike [17]. Of the patients who require surgery, the most commonly fractured bone is the mandible [46]. As a result of this medical necessity, allocating the appropriate funding and resources to physicians is indispensable, and extreme reimbursement cuts may disincentivize surgeons from treating trauma patients in need of emergent care.

Many of the annual percent changes in Table S1 align with the historical progression of the Medicare conversion factor (the changing value used to calculate the physician fee schedule). Out of the 24‐year study period, the largest annual decrease was 9.16%, seen between 2001 and 2002. During this time, there were large policy‐driven reductions in Medicare payments, primarily due to the Sustainable Growth Rate (SGR), leading to a conversion factor cut of 4.8% [47, 48]. This drop is consistent with the sharp decrease in mandibular procedure facility physician reimbursements during the year 2002. Within very little time, though, our results showed an annual change in facility physician reimbursements exhibiting a complete reversal between 2003–2004 and 2004–2005, marked by increases of 8.21% and 8.73%, respectively (Table S1). The 2003 Medicare Prescription, Drug, Improvement, and Modernization Act (MMA) raised funding by 1.5% in 2004 and 2005, directly explaining the reversals we found [48, 49]. Over time, legislation and lobbying have dictated numerous adjustments in physician reimbursement, hence oscillating year‐to‐year percent changes for adjusted facility procedures. Strikingly, the last five straight years have been marked with Medicare physician reimbursement cuts by Congress, causing a spur of contention in the medical community [50, 51].

Medicare's rapid growth over the past two decades has put physician reimbursement at the forefront for cost control in order to balance the federal budget through legislation policies such as SGR. In 2015, Congress repealed SGR and replaced it with the Medicare Access and Children's Health Insurance Program Reauthorization Act (MACRA), designed to increase reimbursement by 0.5% annually [52]. Despite this, physician reimbursement in otolaryngology and otolaryngologic subspecialties continues to decline. With Medicare physician reimbursement significantly lagging behind physician practice expenses, financial pressure on otolaryngologists continues to grow [30, 53]. Reform to the current fee schedule system with alternative payment methods, including Accountable Care Organizations (ACO) and bundled payment systems, often lacks implementation capabilities due to specialty‐specific weaknesses in performance measurement, electronic health infrastructure, and data measurement [54]. More work is needed to create long‐term solutions that are equipped with the necessary tools to sustain adequate reimbursement for physicians.

While our results of facility physician reimbursement have shown steady declines, our analysis of hospital outpatient reimbursement under OPPS revealed a different picture—one marked with an inflation‐adjusted average increase of 110.6% across our six procedures over the past two decades. This sharp disparity between physician and hospital reimbursement was previously noted in analyses of inpatient arthroplasty procedures but has seldom, to our knowledge, been recently explored for outpatient otolaryngologic settings [55, 56]. The distinct technical structures behind MPFS and OPPS calculations may play a role in payment differences, as OPPS is directly tied to the hospital market basket (a measure of growth in hospital costs) and relative APC weights, allowing for built‐in annual recalibrations to combat inflationary pressures [57, 58]. However, further studies are warranted to probe potential misalignments in incentives that underscore divergences in financial trends between physicians and hospitals, which could inherently produce more burden on physicians and distort policy signals.

There are multiple limitations in this study. Data from the CMS Physician Fee Schedule Tool does not consist of information on diagnostic codes or patient demographic data. Modifier impact on fee services also cannot be gauged from the dataset. Hence, no evaluation of epidemiology or procedure complexity was conducted. Moreover, due to public data availability, our timeframes for studying OPPS and MPFS were 20 years and 24 years, respectively, which limit direct comparison in temporal average reimbursement values. Additionally, Medicare reimbursement is only a fraction of reimbursement and does not include private payors or Medicaid, although correlations do exist between Medicare's reimbursement and other insurance programs [29, 59]. Lastly, the assignment of a non‐facility physician fee to a procedure is not indicative of common clinical use in that setting because most of our selected procedures, such as open reduction and internal fixation (ORIF), are overwhelmingly performed in facility environments.

## 5 Conclusion

Inflation‐adjusted facility physician reimbursement under MPFS has steadily declined for mandibular facial fractures between 2000 and 2024. Our findings of an average facility physician reimbursement decrease of 24.1% align with broader trends observed in all types of facial fracture repairs and other otolaryngologic subspecialties. Given the growing elderly patient population, frequency of mandible fractures, declining physician reimbursement in the setting of increasing outpatient hospital reimbursement, and existing barriers to patient access and quality of care, a thorough and continued understanding of Medicare reimbursement trends is imperative. As new legislation and alternative payment models become operational, future studies should explore long‐term solutions that ensure sustainable physician reimbursement structures, particularly for high‐resource, high‐complexity procedures such as open mandibular fracture repair.

## Conflicts of Interest

The authors declare no conflicts of interest.

## Supporting information


**Figure S1:** Unadjusted hospital outpatient procedures for open mandibular fracture procedural codes.

**Figure S2:** Adjusted hospital outpatient procedures for open mandibular fracture procedural codes.

**Figure S3:** Work relative value units (RVUs) for open mandibular fracture procedural codes.

**Table S1:** Average year‐to‐year percent change of selected CPTs from 2000 to 2024 for adjusted physician facility procedures.
**Table S2:** Percent changes in adjusted outpatient prospective payment systems for selected CPT codes between 2000 and 2024
