# Supplementary figures and images for: Medicare Reimbursement Trends for Mandibular Fracture Repair, 2000–2024
Source: Laryngoscope. 2025 Aug 26;136(2):757–65. doi: 10.1002/lary.70084 (PMC12793948; doi:10.1002/lary.70084)

**Fig S3.** Work relative value units (RVUs) for open mandibular fracture procedural codes.

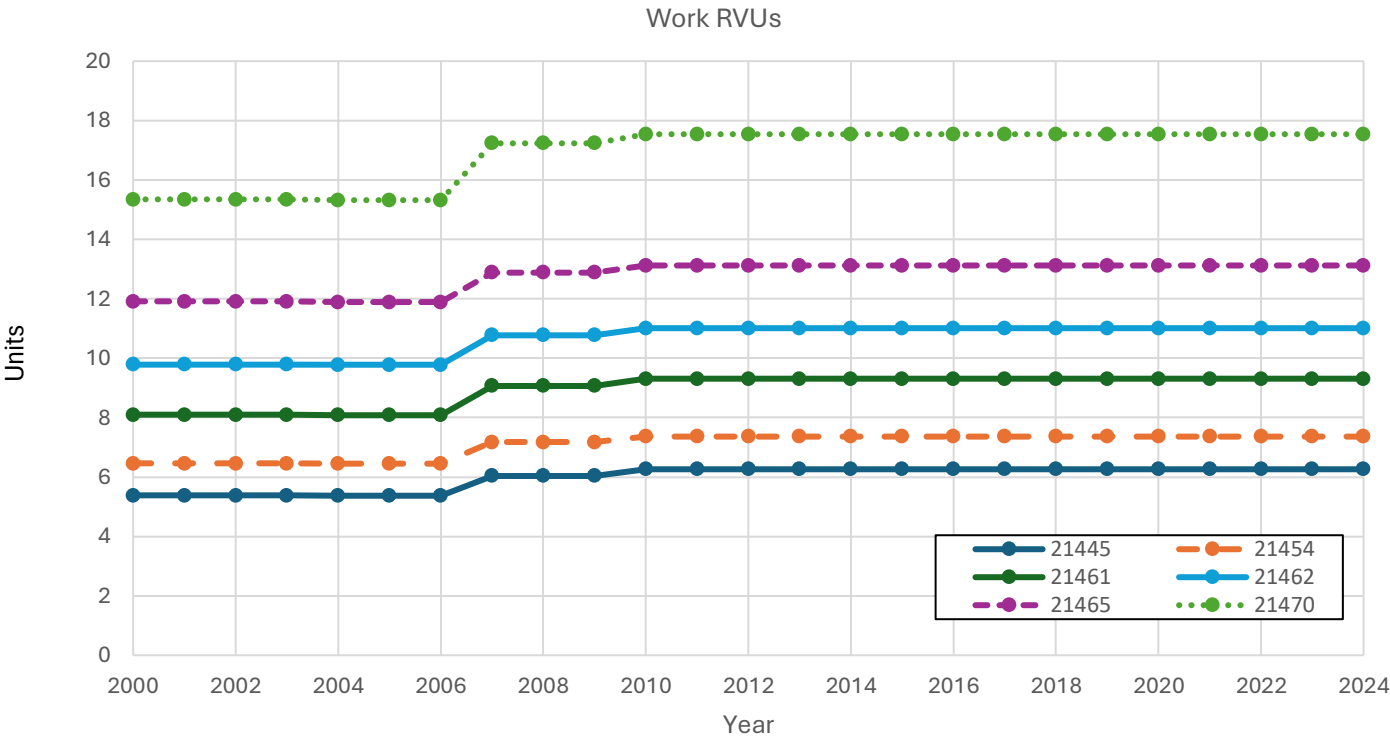

Supplement: Supplementary file 3 — Figure S3: Work relative value units (RVUs) for open mandibular fracture procedural codes. [file LARY-136-757-s001.pdf]
