# Supplementary material for: Medicare Reimbursement Trends for Mandibular Fracture Repair, 2000–2024
Source: Laryngoscope. 2025 Aug 26;136(2):757–65. doi: 10.1002/lary.70084 (PMC12793948; doi:10.1002/lary.70084)
Supplement: Supplementary file 4 — Table S1: Average year‐to‐year percent change of selected CPTs from 2000 to 2024 for adjusted physician facility procedures. Table S2: Percent changes in adjusted outpatient prospective payment systems for selected CPT codes between 2000 and 2024 [file LARY-136-757-s003.docx]

| **TABLE S1.**  ***Average year-to-year percent change of selected CPTs from 2000 to 2024 for adjusted physician facility procedures*** | |
| --- | --- |
| Period | Adjusted Annual Change (%) |
| 2000-2001 | 0.33% |
| 2001-2002 | -9.16% |
| 2002-2003 | -1.44% |
| 2003-2004 | 8.21% |
| 2004-2005 | 8.73% |
| 2005-2006 | -3.33% |
| 2006-2007 | -3.51% |
| 2007-2008 | -5.05% |
| 2008-2009 | 1.16% |
| 2009-2010 | 4.79% |
| 2010-2011 | 2.67% |
| 2011-2012 | -0.48% |
| 2012-2013 | -1.01% |
| 2013-2014 | 1.54% |
| 2014-2015 | -0.80% |
| 2015-2016 | -0.14% |
| 2016-2017 | -2.54% |
| 2017-2018 | -3.03% |
| 2018-2019 | -1.27% |
| 2019-2020 | -2.51% |
| 2020-2021 | -4.66% |
| 2021-2022 | -7.22% |
| 2022-2023 | -5.44% |
| 2023-2024 | -2.64% |
|  |  |
| **AVERAGE** | **-1.12%** |

| **TABLE S2.**  ***Percent changes in adjusted outpatient prospective payment systems for selected CPT codes between 2000 and 2024.*** | | | |
| --- | --- | --- | --- |
| CPT Code | Adjusted Reimbursement Rate in 2004 (in 2024 dollars) | Adjusted Reimbursement Rate in 2024 (in 2024 dollars) | Total Adjusted % Change |
| 21445 | 1983.35 | 5579.71 | 181.33% |
| 21454 | 1983.35 | 5579.71 | 181.33% |
| 21461 | 3185.18 | 5579.71 | 75.18% |
| 21462 | 3185.18 | 5579.71 | 75.18% |
| 21465 | 3185.18 | 5579.71 | 75.18% |
| 21470 | 3185.18 | 5579.71 | 75.18% |
| **AVERAGE** |  |  | 110.56% |
